# Supplementary material for: Microwave assisted antibacterial action of Garcinia nanoparticles on Gram-negative bacteria
Source: Nat Commun. 2022 May 5;13:2461. doi: 10.1038/s41467-022-30125-w (PMC9072325; doi:10.1038/s41467-022-30125-w)
Supplement: Supplementary file 1 — Supplementary information [file 41467_2022_30125_MOESM1_ESM.pdf]

Supplementary Information

Microwave assisted antibacterial action of Garcinia nanoparticles on Gram-negative bacteria

Qiao *et al*.

The PDF file includes:

Supplementary Figs. 1-26

Supplementary Table 1-2

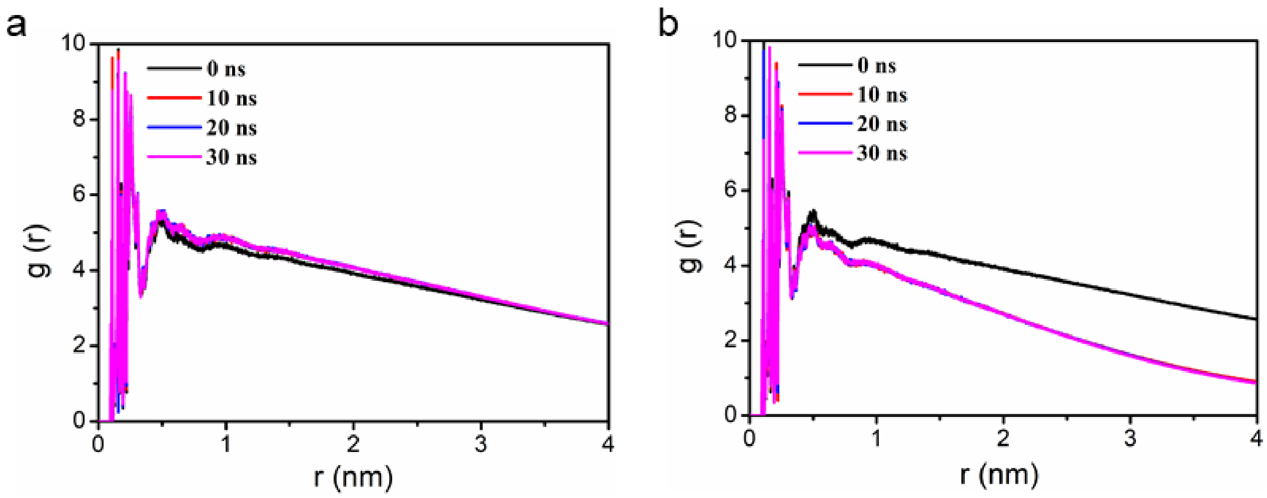

Supplementary Fig. 1 Simulation parameter variation of OM in different groups. Radial distribution function (g (*r*)) change curves of OM at the group of Ctrl (a), and MV (b) within 30 ns. Source data are provided as a Source Data file.

The radial distribution function (g (r)) is used to analyze the orderly changes of OM molecules at short and long distances. In the Ctrl group, as the simulation time progressed, the order of the OM molecules in the short and long distances remained approximately unchanged (Supplementary Fig. 1a), indicating that the overall order of the membrane structure remained unchanged and the structural was relatively stable. On the contrary, the g(r) decreased sharply in the long range ($r \geq 1$ nm) around the OM molecules in the MV group. This suggests that some of the remote OM molecules have diffused beyond the monitoring distance (Supplementary Fig. 1b). The decline of OM structure order is closely related to the formation of membrane pores. The sharp decrease of g (r) further confirms that MV causes OM to form nanopores, which is beneficial to induce nanomedicine enter bacteria.

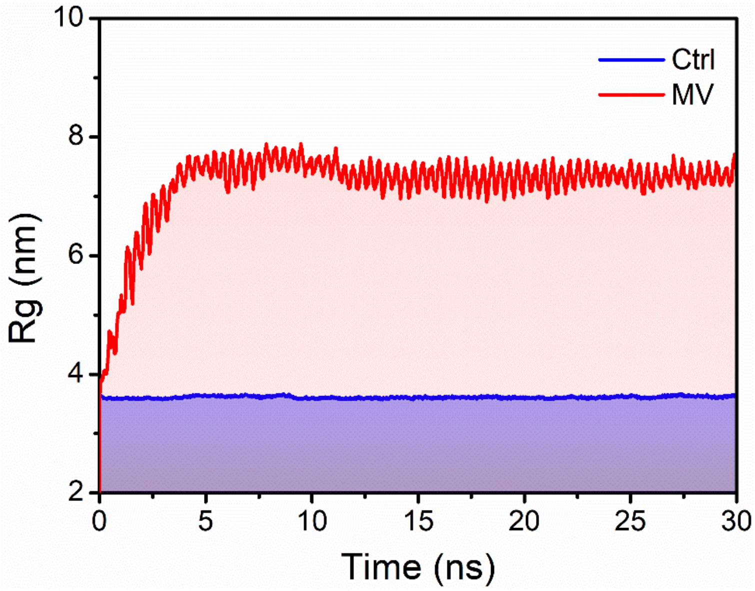

Supplementary Fig. 2 The change of the radius of gyration (R*g*) of the outer membrane in the group of Ctrl and MV. Source data are provided as a Source Data file.

In order to further verify the structural stability of OM, the analysis of the radius of gyration (R*g*) was carried out. The trajectory of the R*g* of the OM can explain the stability of OM in the dynamic process and the tightness of OM structure. As shown in Supplementary Fig. 2, in the Ctrl group, the R*g* of the OM remains about 3.6 nm throughout the dynamic simulation process, suggesting that the OM had high dimensional stability and a hight degree of compactness of aggregated molecular. It could be found that the temperature (55 °C) alone did not cause obviously changes in the OM structure without nanopores. Apparently, in the MV group, the R*g* of OM gradually increased in the initial stage of molecular dynamics simulation (0-10 ns), demonstrate that the OM molecules migrated and the compactness decreased, resulting in a gradual increase in size and the appearance of pores as well as the final formation of nanopores. These results strongly suggest that MV could destroy the tight structure of OM, causing the formation of nanopores in OM, which is beneficial for the entrance of drugs enter the bacteria.

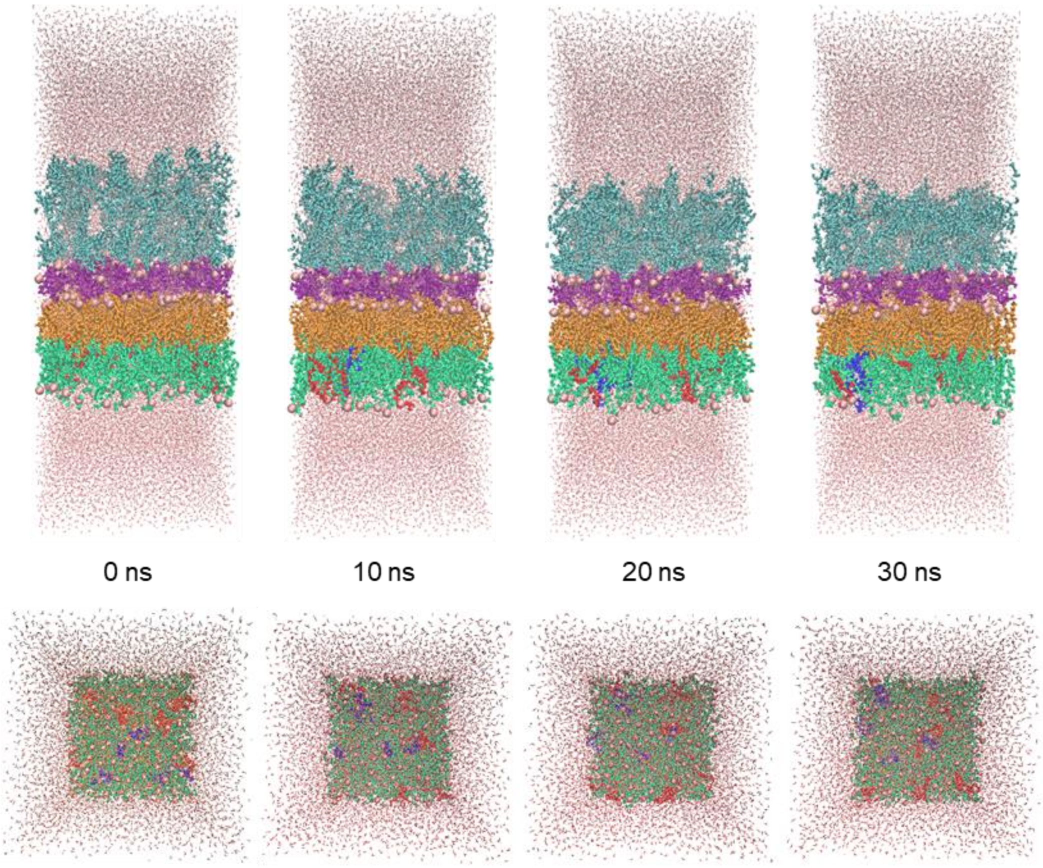

Supplementary Fig. 3 The conformation change of the outer membrane at 55°C (Ctrl) within 30 ns. Front view (above); Bottom view (below).

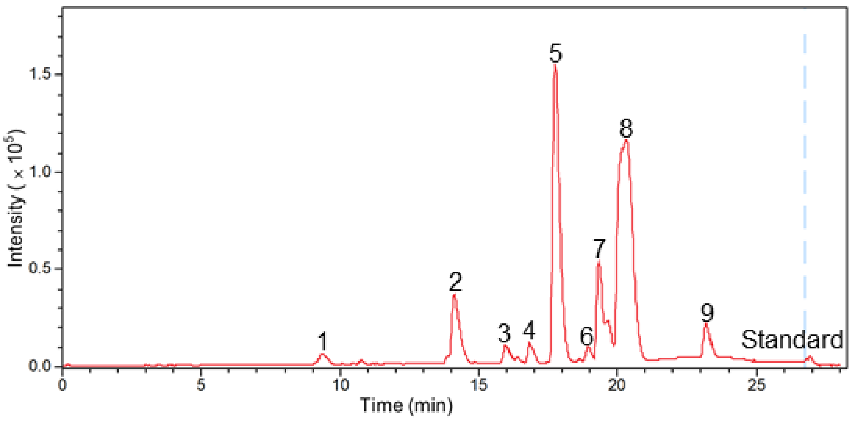

Supplementary Fig. 4 The chromatograms of the GNs.

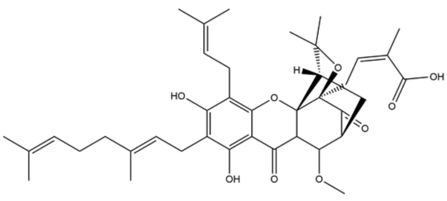

10-methoxygambogenic acid

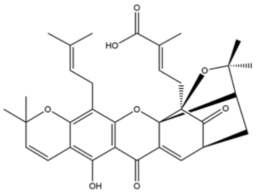

Isomorellic acid

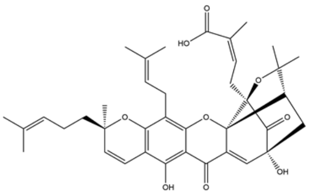

Gambogic acid

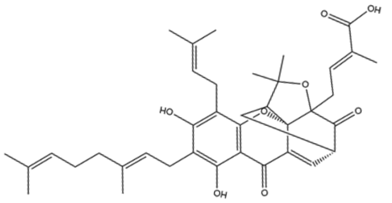

Isogambogenic acid

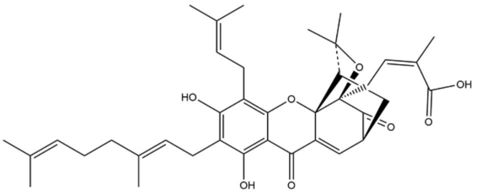

Gambogenic acid

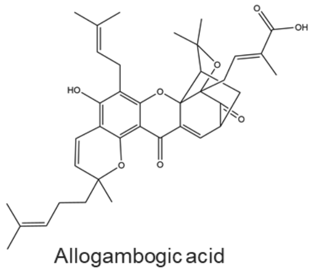

Allogambogic acid

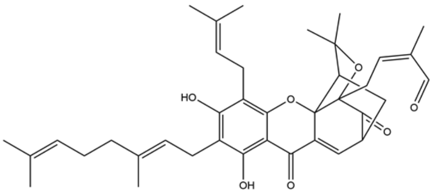

Isogambogenin

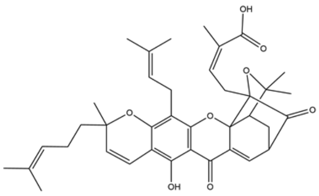

α-Gambogic acid

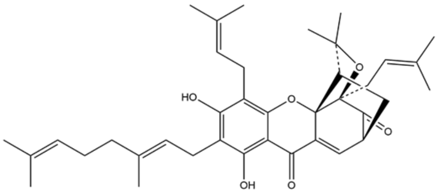

Desoxygambogenin

Supplementary Fig. 5. **The chemical structures of the nine molecules contained in GNs.**

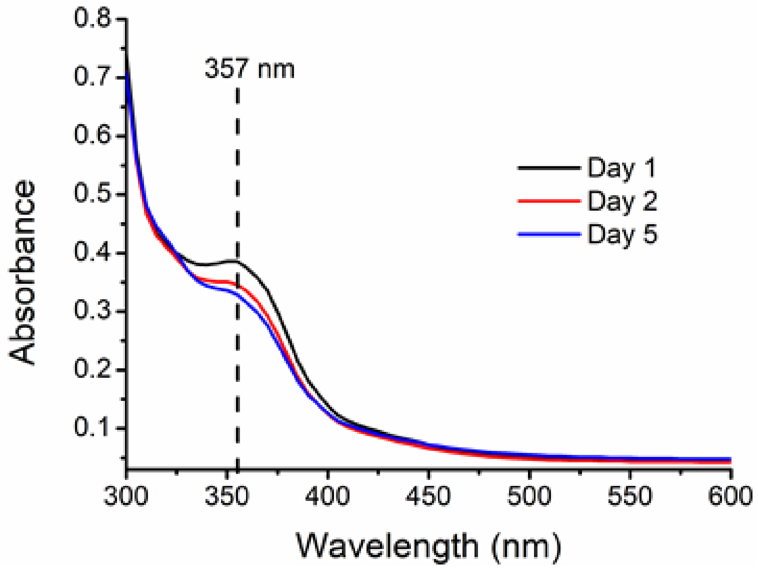

Supplementary Fig. 6. **Degradation absorbance curves of GNs.** Source data are provided as a Source Data file.

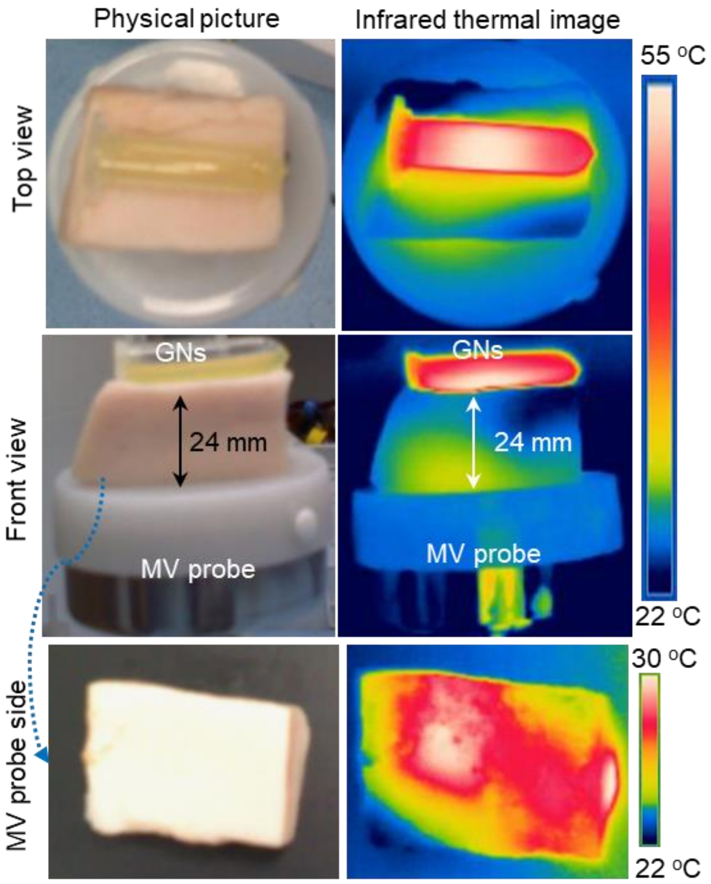

Supplementary Fig. 7 Infrared thermal images of GNs through 24 mm pork tissue under MV excitation.

We increased the thickness of the pork to 24 mm, and the GNs solution can still be heated to 55°C (Supplementary Fig. 7). Furthermore, through the infrared thermal images of the front view, the top view, and the tissue next to the probe, it can be seen that the tissue temperature has not increased significantly (about 30°C). These results illustrated that the GNs solution can achieve desired MV thermal performance even at 24 mm penetration depth without causing significant heating inside the tissues.

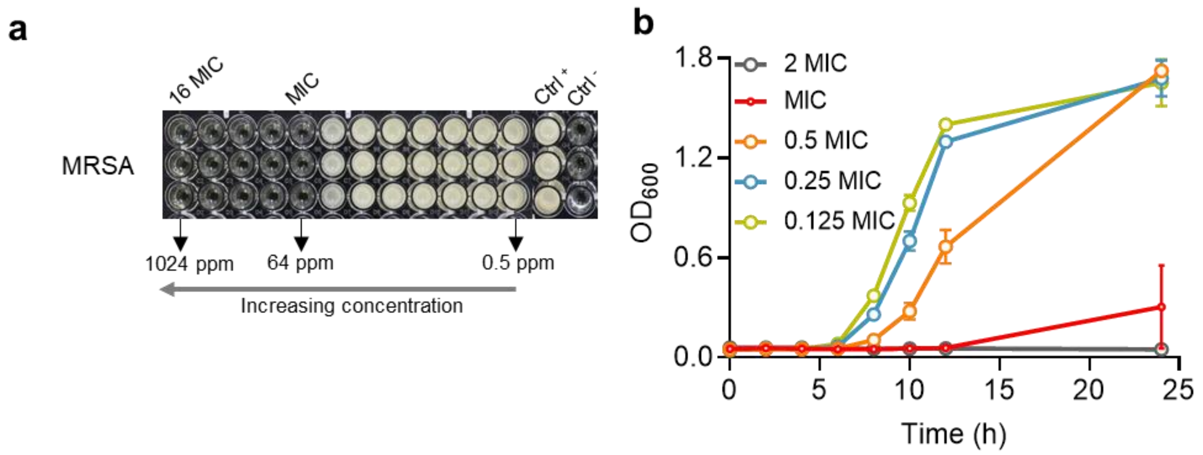

Supplementary Fig. 8 Antibacterial effect of GNs against MRSA. a, The MIC test of GNs against MRSA. **b,** The growth curves of MRSA with different GNs concentrations. Data are presented as mean ± standard deviations from a representative experiment (*n* = 3 independent samples). Source data are provided as a Source Data file.

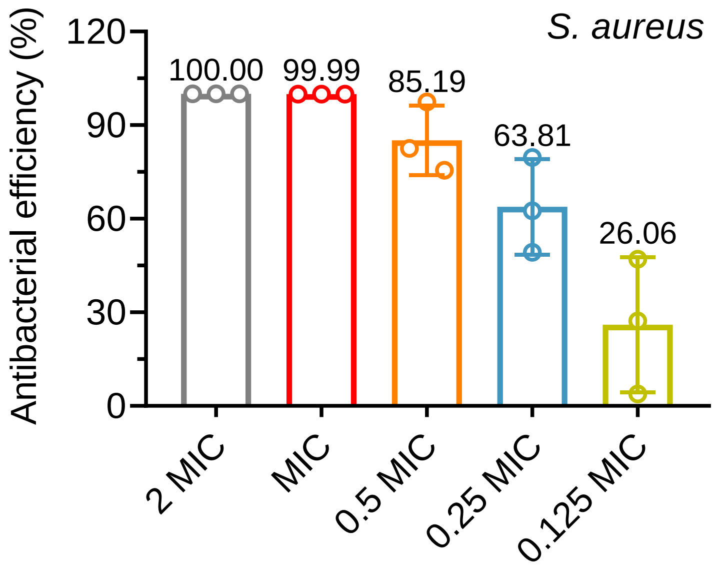

Supplementary Fig. 9 Statistics results of the antibacterial ability of GNs against *S. aureus*. Data are presented as mean ± standard deviations from a representative experiment (*n* = 3 independent samples). Source data are provided as a Source Data file.

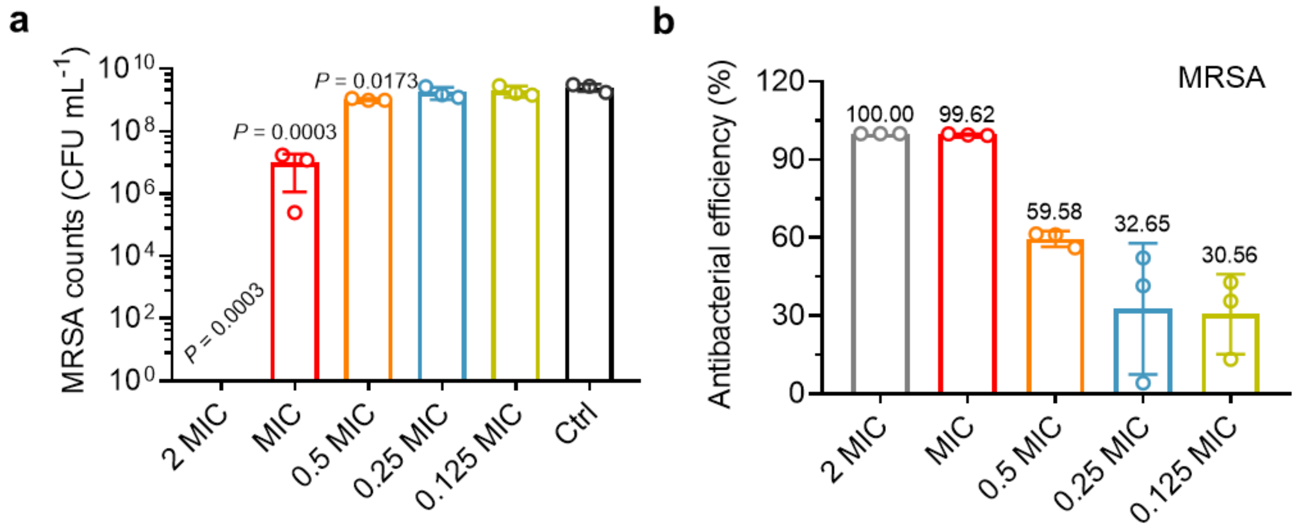

Supplementary Fig. 10 Antibacterial effect of GNs against MRSA. a, MRSA strain counts calculated from spread-plate assays after treatment with different concentrations of GNs. **b,** Statistics results of the antibacterial ability of MRSA. Data are presented as mean ± standard deviations from a representative experiment (n = 3 independent samples for **a** and **b**). P values were analyzed by one-way ANOVA with Dunnett's multiple comparisons post hoc test. Source data are provided as a Source Data file.

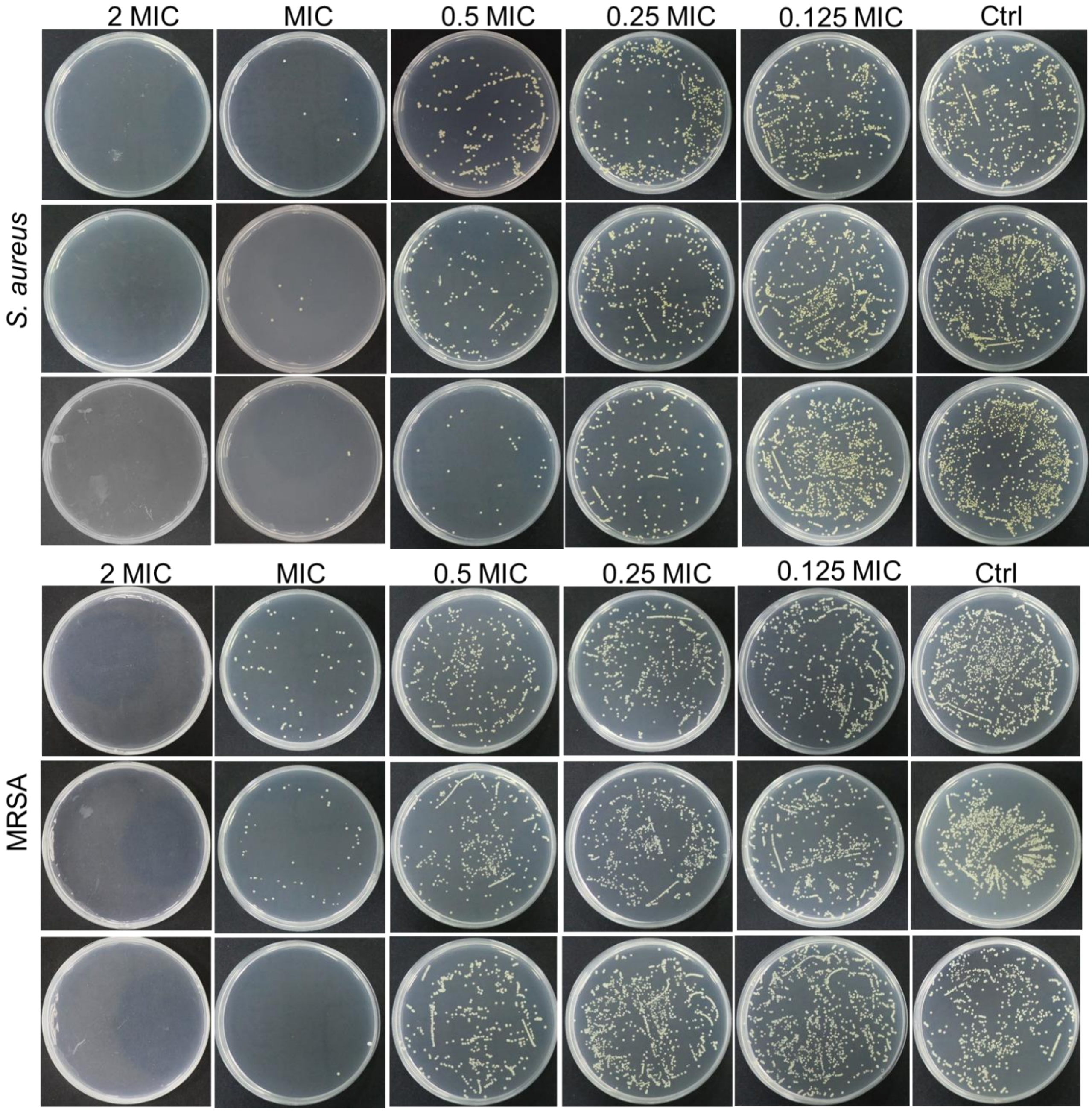

Supplementary Fig. 11 In vitro antibacterial test by spread plate method. *S. aureus* or MRSA after 16 hours of co-culture with different concentrations of GNs (Ctrl, 0.125 MIC, 0.25 MIC, 0.5 MIC, MIC, and 2 MIC) spread onto LB agar plates and incubated at 37 °C for 20 hours.

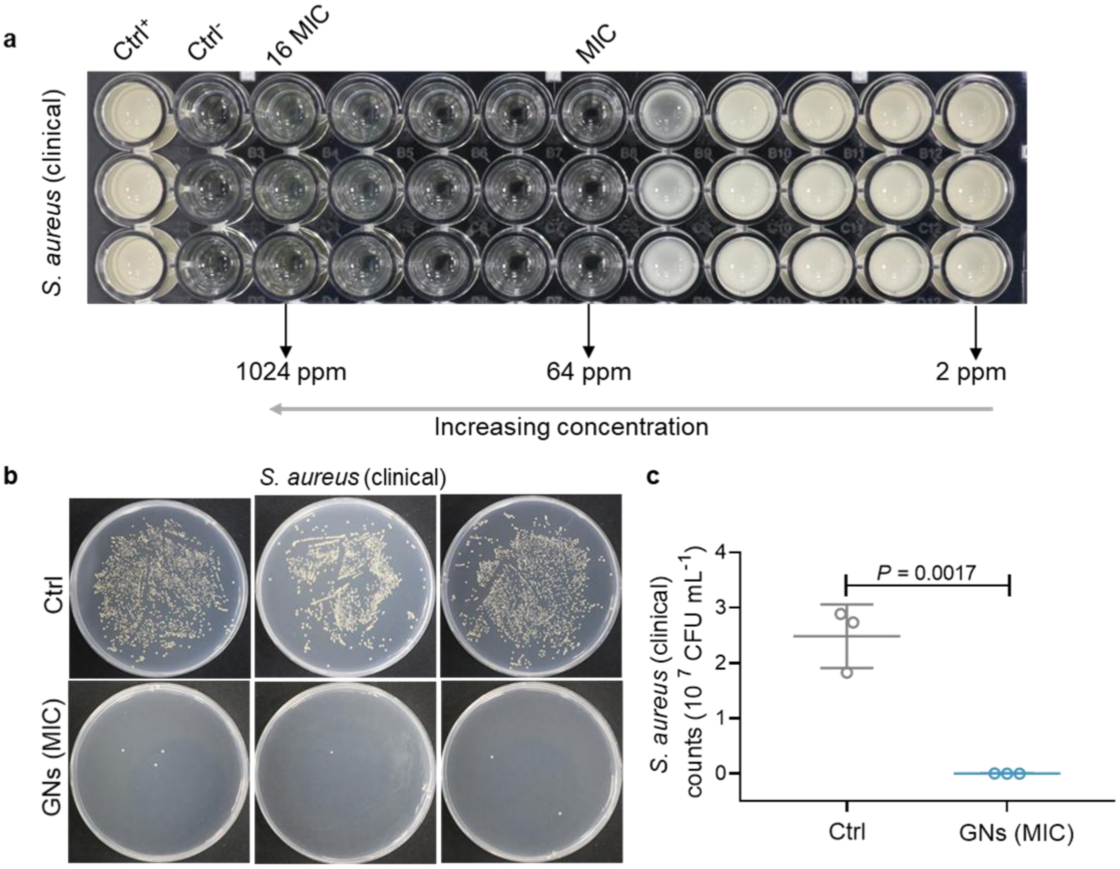

Supplementary Fig. 12 GNs efficiently eradicate *S. aureus* (clinical) strains. a, The MIC test of GNs on clinical *S. aureus*. **b,** GNs against *S. aureus* (clinical) tested by spread plate method. **c,** *S. aureus* (clinical) strain counts calculated from spread-plate assays after GNs treated. Data are presented as mean ± standard deviations from a representative experiment (n = 3 independent samples for **c**). P value was analysed by unpaired t test. Source data are provided as a Source Data file.

The antibacterial tests revealed that the MIC of GNs against clinical *S. aureus* is 64 ppm (Supplementary Fig. 12a). Furthermore, the antibacterial effect of GNs against clinical *S. aureus* strains was also confirmed by the spread-plate assay (Supplementary Fig. 12b). Specifically, the clinical *S. aureus* counts were obviously reduced to 0.002 (10^7 CFU mL^{-1}) in the GNs (MIC) group with 99.92% antibacterial rate (Supplementary Fig. 12c).

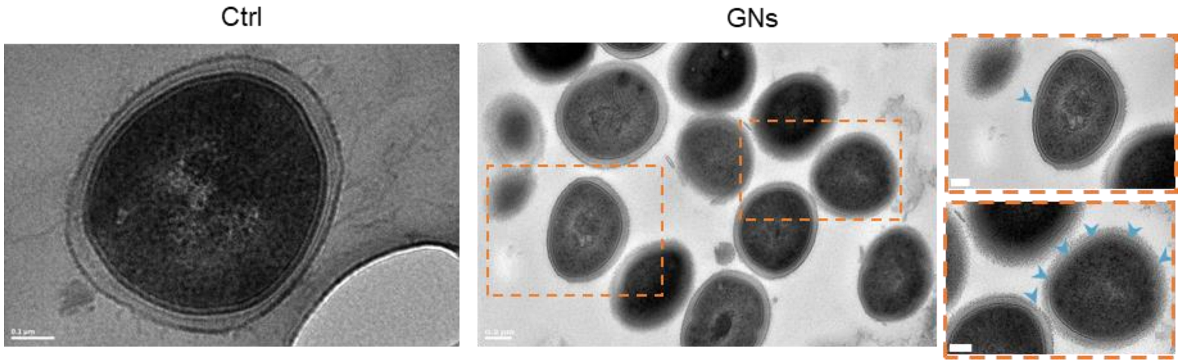

Supplementary Fig. 13 Representative TEM images of *S. aureus* treated with GNs for 8 hours. Scale bar, 100 nm (enlarged view).

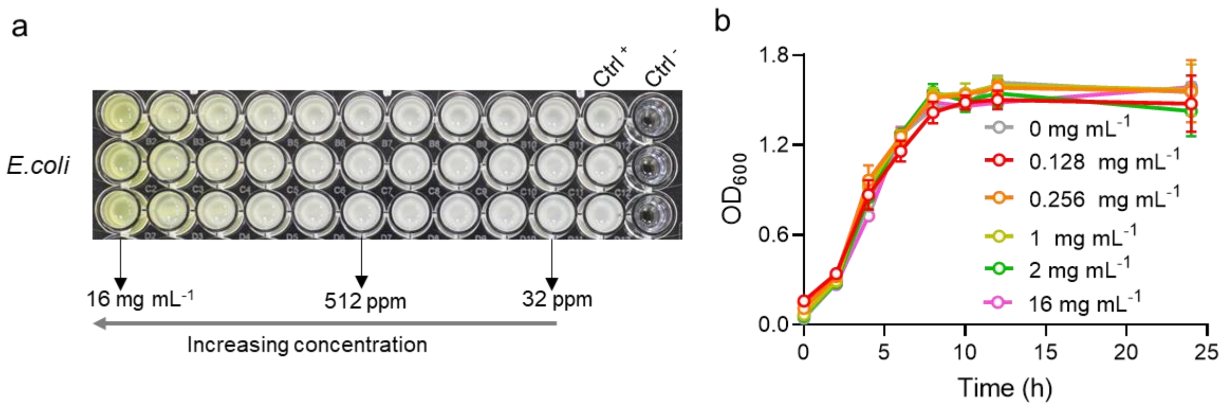

Supplementary Fig. 14 GNs have low activity against *E. coli*. a, The MIC test of GNs against *E. coli*. **b,** The growth curve of *E. coli* at different GNs concentrations. Data are presented as mean ± standard deviations from a representative experiment ($n = 3$ independent samples). Source data are provided as a Source Data file.

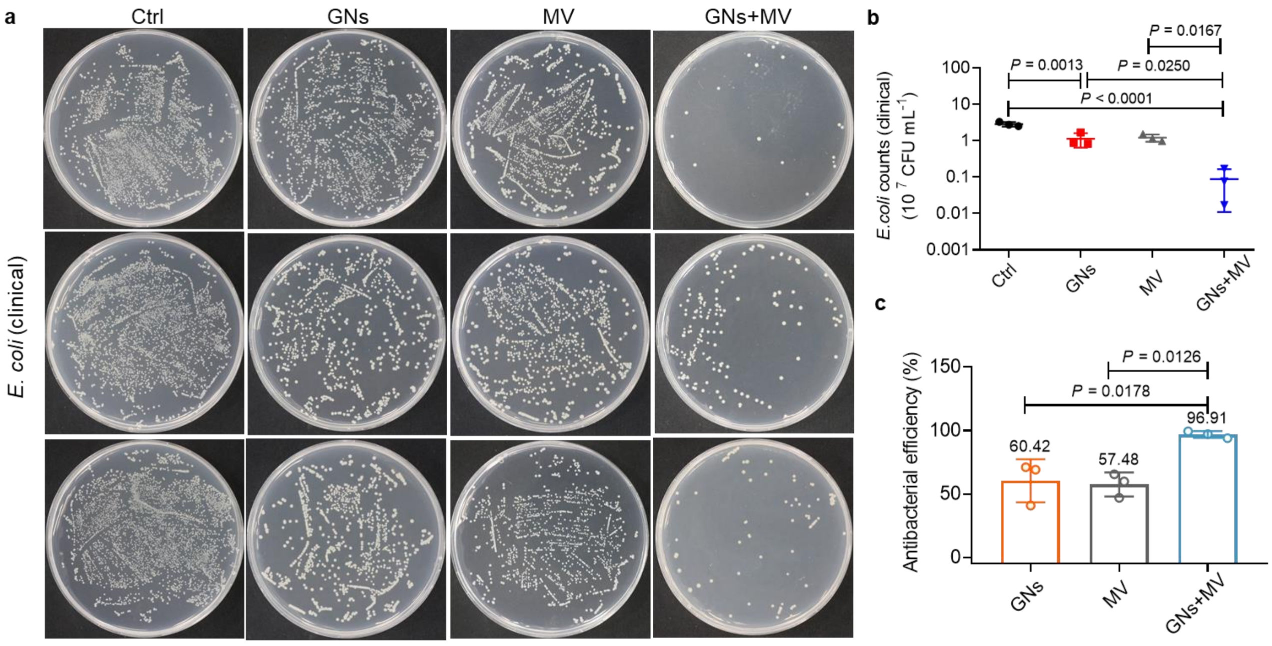

Supplementary Fig. 15 The efficacy of GNs and MV synergistically killing clinical *E. coli* strains. a, GNs against clinical *E. coli* under MV or not tested by spread plate method. **b**, Clinical *E. coli* strain counts calculated from spread-plate assays after treatment with GNs under MV excitation for 15 minutes or not. **c,** Statistics results of the antibacterial ability of the clinical *E. coli*. Data are presented as mean ± standard deviations from a representative experiment (*n* = 3 independent samples for **b** and **c**). P values were analysed by one-way ANOVA with Tukey's multiple comparisons post hoc test. Source data are provided as a Source Data file.

GNs (4 mg mL^{-1}) has a weak antibacterial effect on *E. coli* (clinical). Notably, the antibacterial effect is significantly improved after synergistic MV treatment (Supplementary Fig. 15a). Specifically, *E. coli* (clinical) counts (10^7 CFU mL^{-1}) in the GNs + MV group average fell to 0.088 (P <0.0001, compared to Ctrl), lower than other groups (Supplementary Fig. 15b), demonstrating that 96.91% *E. coli* (clinical) were killed by GNs during 15 minutes of MV treatment (Supplementary Fig. 15c). In contrast, the antibacterial rate of GNs and MV alone was 60.42% and 57.48%, respectively (Supplementary Fig. 15c), far lower than that of the group of GNs + MV.

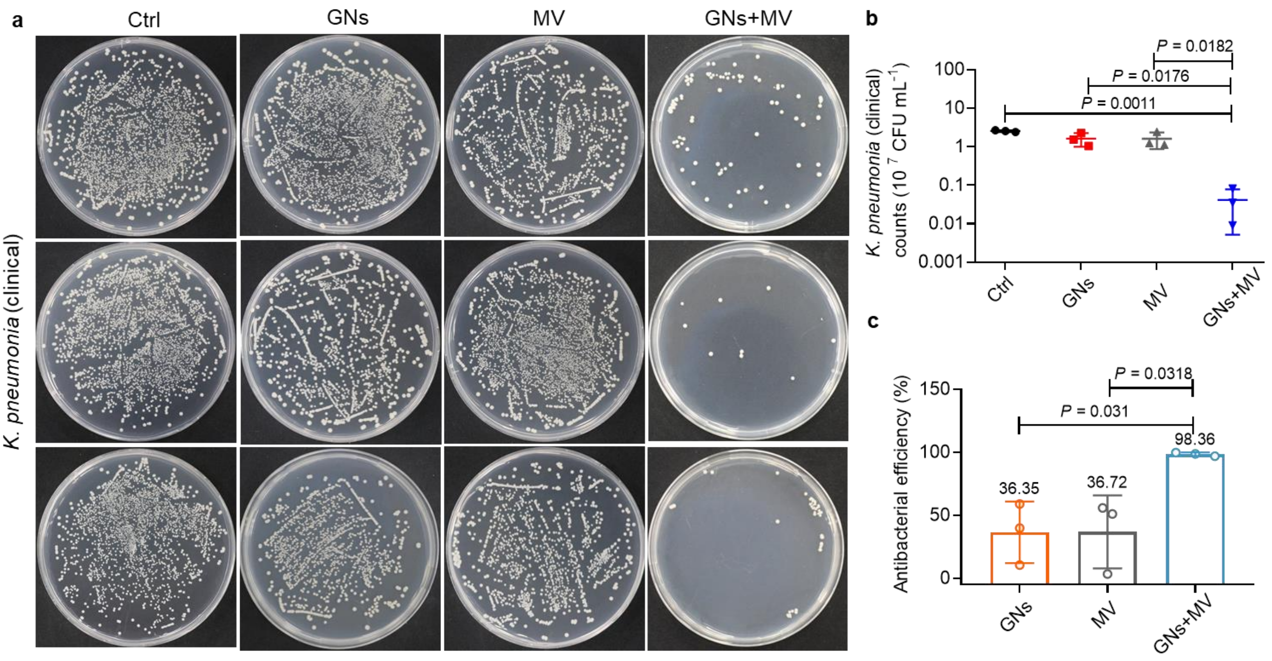

Supplementary Fig. 16 The efficacy of GNs and MV synergistically killing clinical multi-drug resistant *K. pneumonia* strains. a, GNs against clinical multi-drug resistant *K. pneumonia* under MV or not tested by spread plate method. **b**, Clinical multi-drug resistant *K. pneumonia* strain counts calculated from spread-plate assays after treatment with GNs under MV excitation for 15 minutes or not. **c,** Statistics results of the antibacterial ability of the clinical multi-drug resistant *K. pneumonia*. Data are presented as mean ± standard deviations from a representative experiment (n = 3 independent samples for **b** and **c**). P values were analysed by one-way ANOVA with Tukey's multiple comparisons post hoc test. Source data are provided as a Source Data file.

Similarly, GNs (4 mg mL^{-1}) has a weak antibacterial effect on multi-drug resistant *K. pneumonia* strains (clinical), and the antibacterial effect is significantly improved after synergistic microwave treatment (Supplementary Fig. 16a). Specifically, multi-drug resistant *K. pneumonia* strains (clinical) counts (10^7 CFU mL^{-1}) in the GNs + MV group average fell to 0.042, lower than other groups (Supplementary Fig. 16b), demonstrating that 98.36% *K. pneumonia* (clinical) were killed by GNs during 15 minutes of MV treatment (Supplementary Fig. 16c). In contrast, the antibacterial rate of GNs and MV alone was 36.35% and 36.72%, respectively (Supplementary Fig. 16c), far lower than that of the group of GNs + MV.

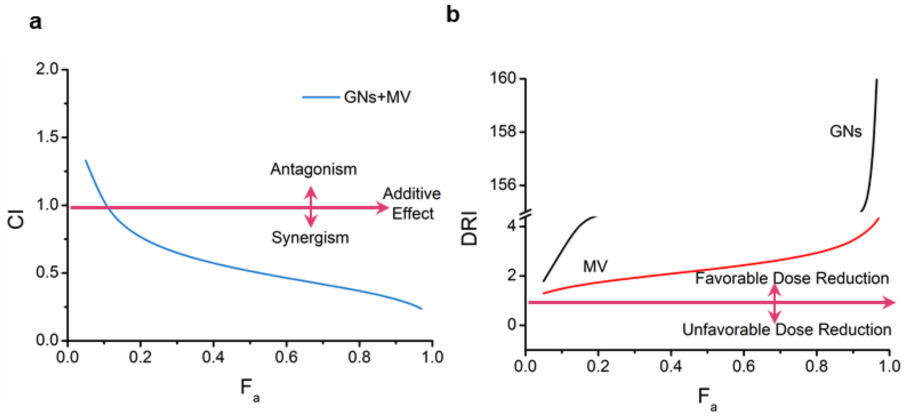

Supplementary Fig. 17 Parameters related to the synergy between GNs and MV. a, Fa-CI plot. **b,** Fa-DRI plot.

Source data are provided as a Source Data file.

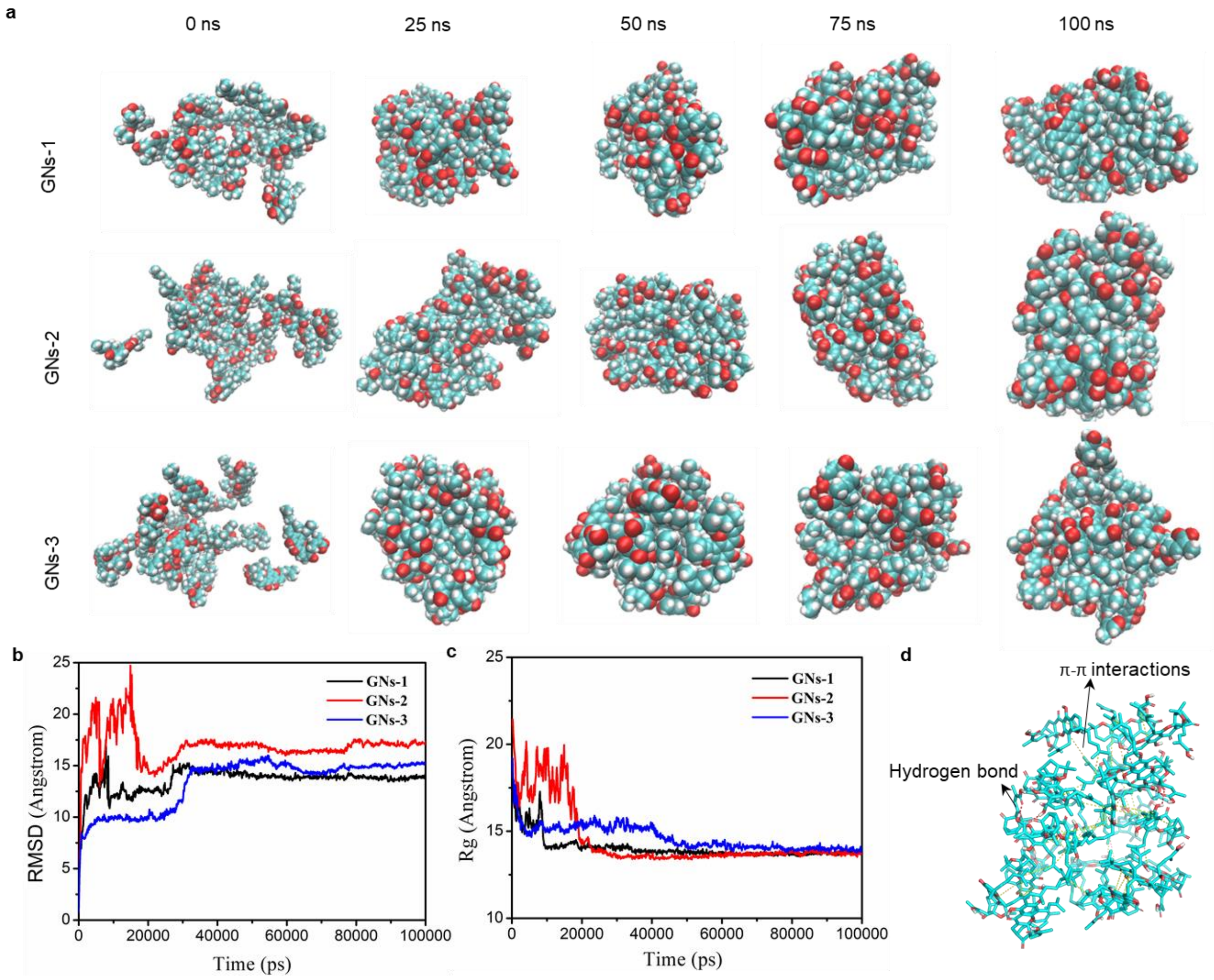

Supplementary Fig. 18 GNs stability analysis. a, Representative configurations of molecular dynamics simulation of GNs in top 3 terms by molecular docking at 25, 50, 75, and 100 ns. Atomic color coding in crystal structure: C-cyan, O-red, and H-light grey. **b**, **c,** RMSD (**b**) and Rg (**c**) of these models in top three terms to evaluate their stability by MD simulation (100 ns). **d,** Specific model of GNs-1 by hydrogen bonds (red lines) and π-π interactions (yellow lines). Source data are provided as a Source Data file.

We selected five monomers with higher content in GNs as the main components for constructing nanoparticles, and the number of monomer molecules in GNS was based on their mass ratio (α-Gambogic acid 10, Gambogenic acid 7, Isogambogenin 2, Isomorellic acid 2, and Desoxygambogenin 1). Three small-molecule dispersion systems of GNs (GNs-1, GNs-2, and GNs-3) were constructed using the Packmol program. To study the stability of GNs in solvent systems, 100 ns molecular dynamics simulations were performed for these three GNs. As shown in supplementary Fig. 18 a, during the 100 ns simulation, the structure of GNs-1 remained compact and

stable. On the contrary, the conformational changes of GNs-2 and GNs-3 are more obvious, and the sharpening of the surface has the risk of small molecules falling off (100 ns). In addition, the RMSD values (supplementary Fig. 18 b) of the three GNs fluctuated greatly in the first 30 ns of the simulation, which was due to the gradual migration and aggregation of the five monomers from the dispersed state, and the self-assembly to form nanoparticles. After 30 ns, the RMSD value fluctuated less and tended to equilibrium, indicating that the assembled nanoparticles tended to be stable in the solvent system. It is worth noting that the fluctuation of the RMSD value of GNs-1 after system equilibrium is less than 1 Å, while the fluctuation of GNs-2 and GNs-3 is close to 3 Å, indicating that the stability of GNS-1 is better than that of GNs-2 and GNs-3. Besides, Rg (supplementary Fig. 18 c) also showed a similar trend. GNs-1 possessed definite stability due to their abundant hydrogen bonds (red lines) , strong p-p interactions (dotted yellow lines), and hydrophobic interactions on alkene branches (supplementary Fig. 18 d). in These results demonstrate that GNs-1 has the most stable structure for subsequent MV-assisted transmembrane studies.

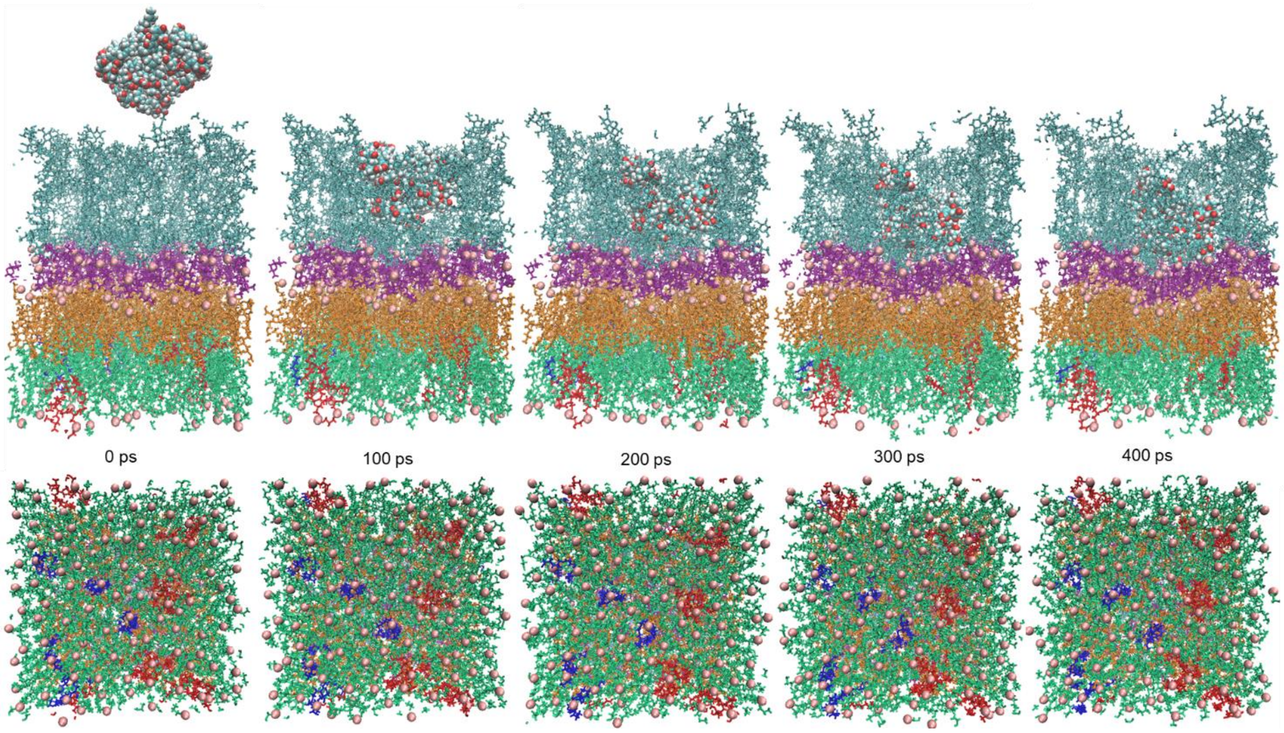

Supplementary Fig. 19 Dynamic behavior of GNs-1 during tensile dynamics simulation without MV.

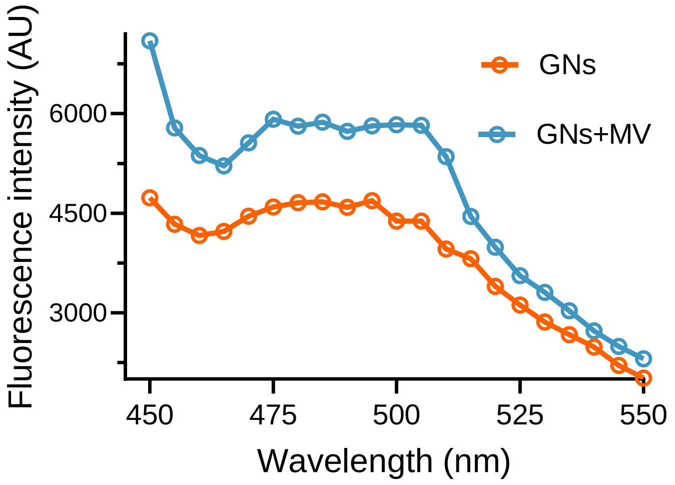

Supplementary Fig. 20 OM permeability test using ANS. Source data are provided as a Source Data file.

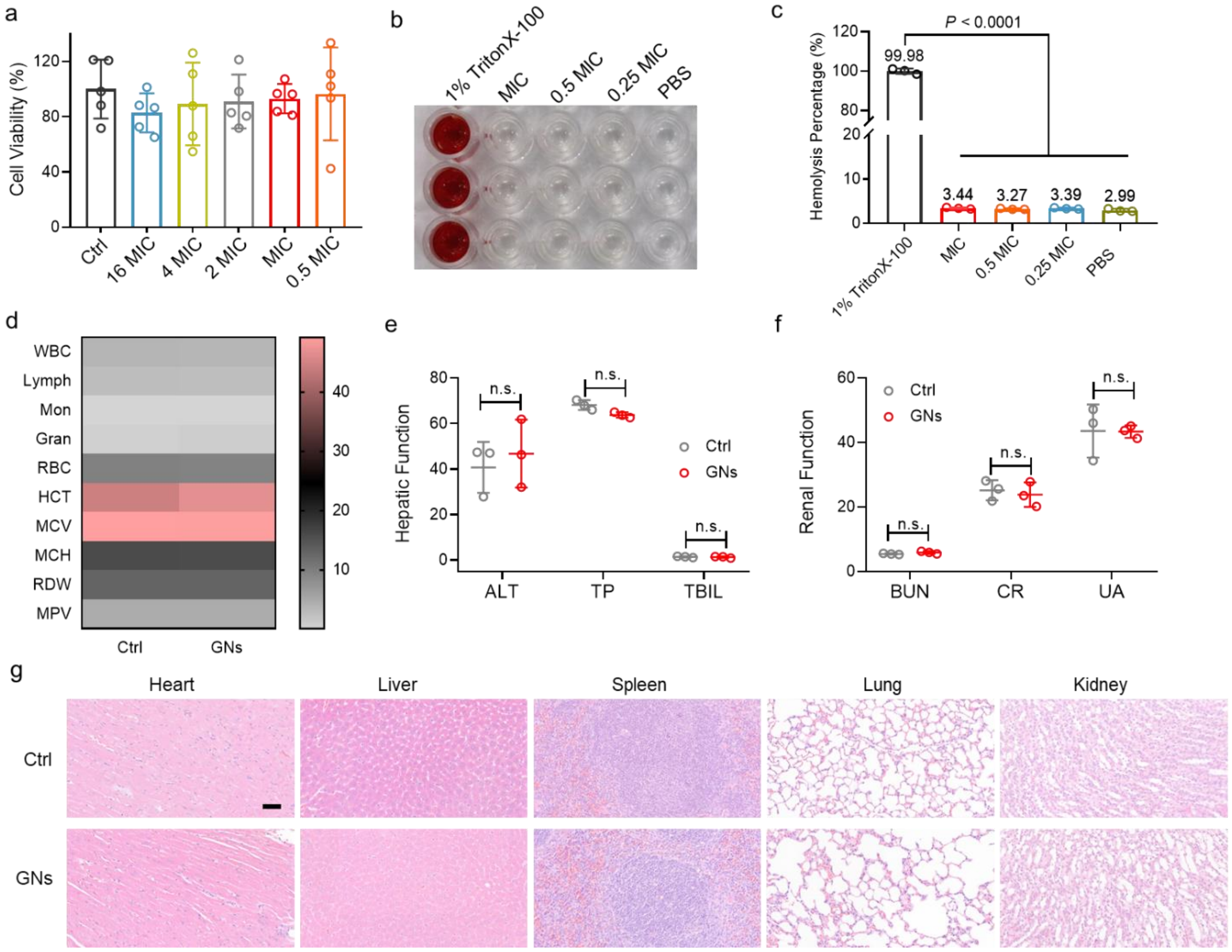

Supplementary Fig. 21 In vitro and vivo safety evaluation. a, The viability of NIH-3T3 cells cocultured with different concentrations of GNs for three days. **b,** Hemolysis images of different concentrations of GNs. **c,** Hemolytic efficiency of different concentrations of GNs. **d,** Parameters of complete blood tests of mice after post-injection of GNs at 7 days. **e,** Hepatic function (ALT, TP, and TBIL) of Ctrl and GNs groups on day 7. **f,** Renal function (BUN, CR, and UA) of Ctrl and GNs groups on day 7. **g,** Representative H&E staining images of internal organs injury in the given dose GNs. Scale bar, 50 μm. Data are presented as mean ± standard deviations from a representative experiment ($n = 5$ independent samples for **a**,**d**; $n = 3$ independent samples for **c, e, f**. The n.s. present $P > 0.05$, and P values were analysed by two-way ANOVA with Tukey's multiple comparisons post hoc test for **c,** and P values were analysed by two-way ANOVA with Sidak's multiple comparisons post hoc test for **e, f.** WBC, white blood cells; Lymph, number of lymphocytes; Mon, monocyte; Gran, granulocyte; RBC, red blood cells; HCT, hematocrit; MCV, mean red blood cell volume; MCH, mean erythrocyte hemoglobin; RDW, red blood cell distribution width. MPV, mean platelet volume; ALT, alanine transaminase; TP, total protein; TBIL, total bilirubin; BUN, blood urea nitrogen; CR, creatinine; and UA, uric acid. Source data are provided as a Source Data file.

In vitro and vivo safety evaluation.

To verify GNs as a safe and effective approach for bacterial infection therapy, the cellular toxicity of GNs was assessed using NIH-3T3 fibroblasts. The cytotoxicity of GNs was assessed using the MTT assay (Supplementary Fig. 21a). After three days of coculturing, compared with the control group, the groups of 0.25 MIC, MIC, 2 MIC, 4MIC and even 16 MIC showed a cell viability of over 80%. This revealed that GNs had excellent cytocompatibility. After that, the blood safety of GNs was evaluated by measuring the hemolysis percentage of different concentrations of GNs. As shown in Supplementary Fig. 21b, the red blood cells in 1% TritonX-100 (the positive control) group were ruptured, and consequently the solution became red. As a contrast, the solutions of 0.25 MIC, 0.5 MIC and MIC groups were clarified and did not change in color compared to PBS (negative control group), demonstrate that hemolysis did not occur. Meanwhile, the hemolytic percentage of all test concentrations of GNs in this experiment were as low as the PBS, and far lower than that of the positive control (Supplementary Fig. 21c), indicating the good blood compatibility of GNs.

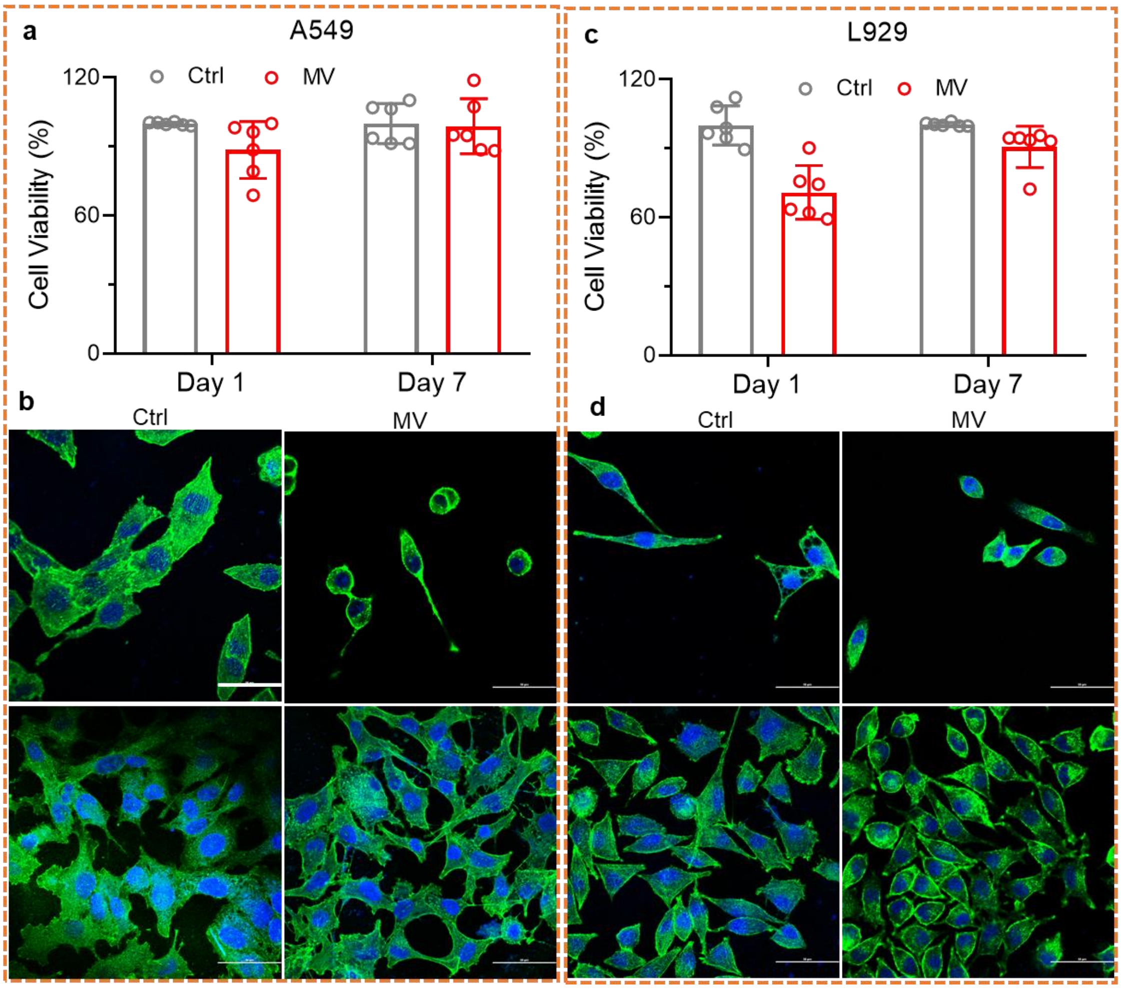

Supplementary Fig. 22 The effect of MV irradiation on the viability and morphology of normal cells. a, c, The viability of A549 (**a**) and L929 (**c**) were cultured for one and seven days after treatment of MV or not. n = 6 independent samples for **a** and **c**. **b, d,** Fluorescent images of A549 (**b**) and L929 (**d**) were cultured for one and seven days after treatment of MV or not. Scale bar, 50 μm. Data are presented as mean ± standard deviations from a representative experiment (*n* = 6 independent samples for **a** and **c**). Source data are provided as a Source Data file.

Then we studied the effects of microwave irradiation on the proliferation and morphology of normal cells. As show in Supplementary Fig. 22, after MV treatment for one day the viability of A549 and L929 cells decreased to 88.56% (Supplementary Fig. 22 a) and 70.89% (Supplementary Fig. 22b), respectively. And the cell viability was restored to 98.83% (Supplementary Fig. 22 a) and 90.64% (Supplementary Fig. 22b), respectively, after continuing the culture to the seventh day. Similarly, after MV treatment for one day, the morphology of A549 and L929 cells changed from a polygonal shape with filamentous pseudopodia to a fusiform antenna that shortened and shrank, and the cells shrank into a spherical structure (Supplementary Fig. 22b,d). Continue to culture to the seventh day, the cell

morphology returned to normal, and the number of cells increased (Supplementary Fig. 22b,d). These results indicate that cell proliferation will be inhibited after MV treatment, but after a short period of recovery, the cells can gradually recover and begin to proliferate.

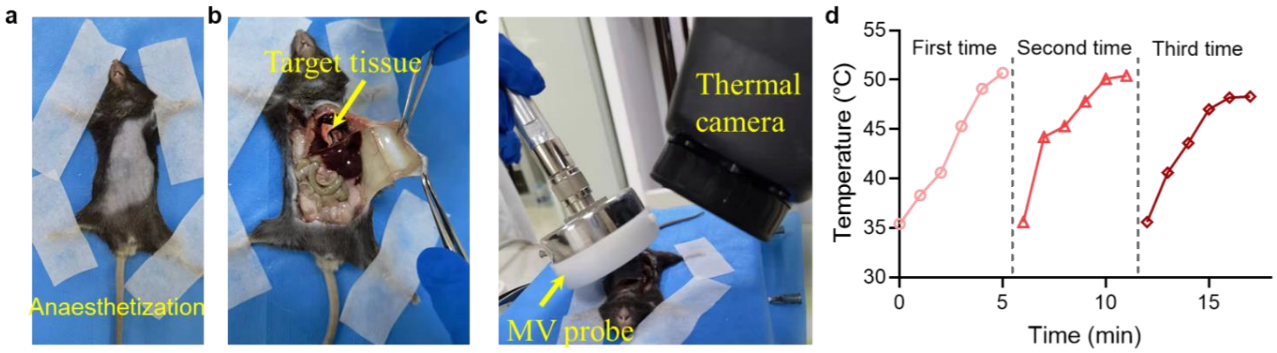

Supplementary Fig. 23 Experimental procedures for detecting the temperature of mouse lungs during treatment. a-c, Experimental operation steps; d, Temperature changes in the lungs of mice during treatment. Source data are provided as a Source Data file.

Specifically, the mice were anesthetized after inhaling the aerosolized GNs and shaved the abdominal hair, and then cut the skin on the side of the mouse's abdomen. The purpose is to uncover the skin of the mouse abdomen after the target tissue is irradiated by a microwave probe to facilitate the thermal camera to record the temperature of the lungs. The operation process of microwave therapy is shown in Supplementary Fig. 23a-c. The actual temperature of the lungs in the mouse is shown in Supplementary Fig. 23d. With the extension of the microwave irradiation time, the temperature of the lungs gradually increased and reached 50°C within five minutes.

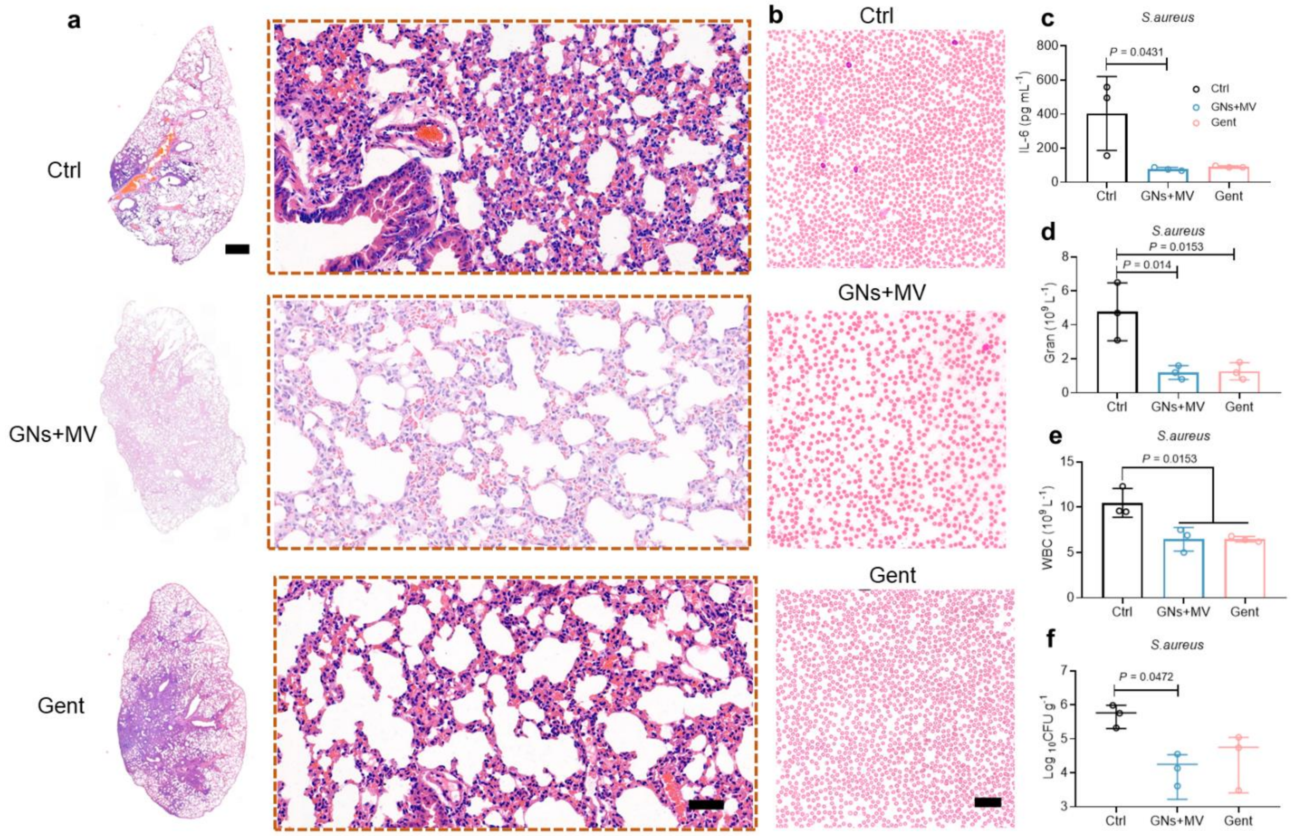

Supplementary Fig. 24. Antibacterial effects of GNs on *S. aureus* monoinfect pneumonia in vivo. a, Representative H&E staining images of infected lung tissues after 7 days of treatment. Scale bars, 1 mm and 50 µm (enlarged view). **b,** Representative Wright-stained images of blood in mice with *S. aureus* monoinfect after one day of treatment. Scale bars, 20 µm. **c-f**, IL-6 levels (**c**), amount of Gran (**d**) and amount of WBC (**e**) for 1 day in blood. **f**, Bacteria counts in the infected lung after one day with different treatments. Data are presented as mean ± standard deviations from a representative experiment ($n = 3$ biologically independent samples). P values were analysed by two-way ANOVA with Tukey's multiple comparisons post hoc test. Source data are provided as a Source Data file.

The panoramic view of the HE slice in the mouse model of *S.aureus* monoinfect pneumonia we can see that there is a large area of focal infiltration of pink mucus inflammatory cells, and the enlarged image clearly shows the proliferation of alveolar epithelial cells (Supplementary Fig. 24a). In contrast, there were no obvious symptoms of infection in the GN+MV and Gent groups. Similarly, the Wright-stained blood samples in the treatment group significantly reduced the number of lymphocytes (Supplementary Fig. 24b). And the IL-6 (Supplementary Fig. 24c), Gran (Supplementary Fig. 24d), and WBC (Supplementary Fig. 24e) levels in the GNs +MV and Gent group were significantly lower than that in Ctrl group, indicating that the bacterial infection was restrained in the treatment groups.

In addition, the number of *S. aureus* in the lungs was significantly reduced in the treatment groups (Supplementary Fig. 24f). Notably, the number of bacteria in the GN+MV group was lower than that in the antibiotic group. These results fully prove the excellent performance of GN+MV in the treatment of pneumonia caused by *S. aureus*.

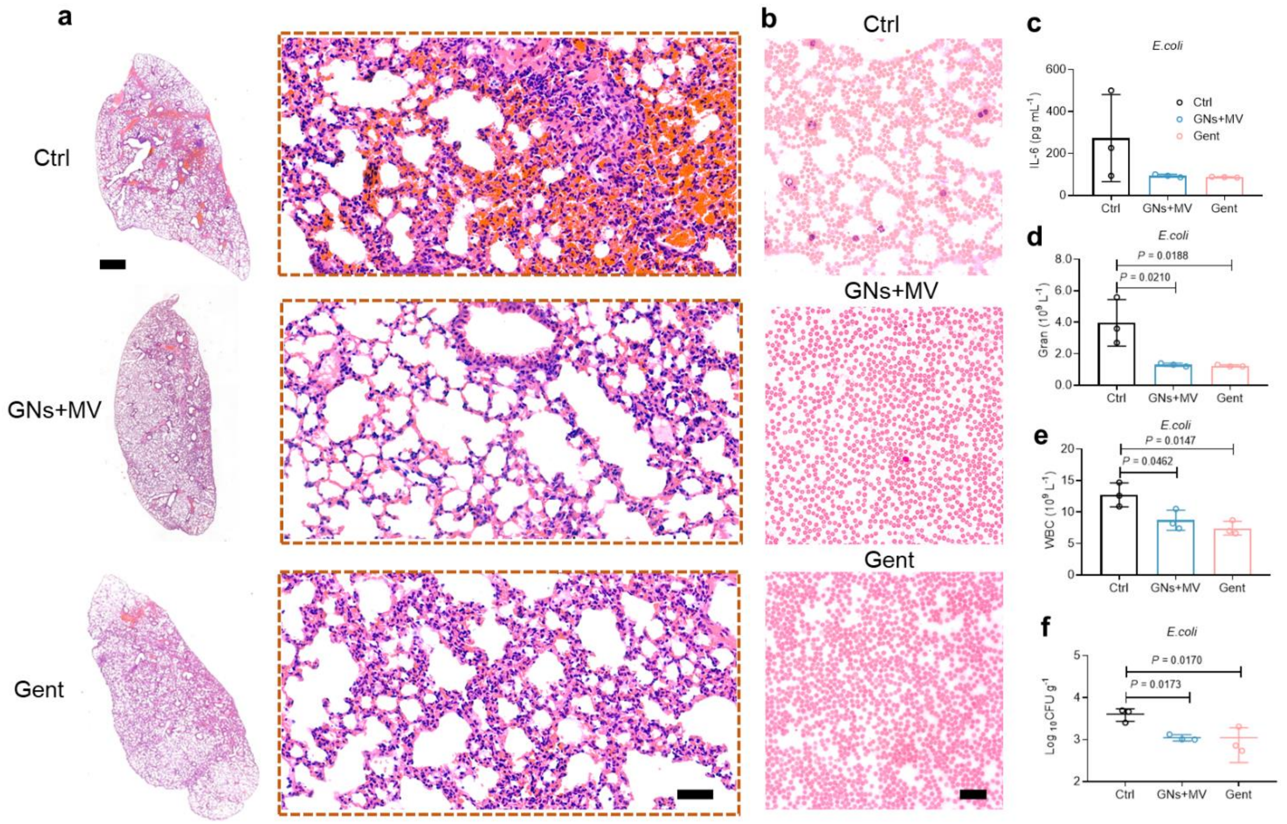

Supplementary Fig. 25 Antibacterial effects of GNs on *E. coli* monoinfect pneumonia in vivo. a, Representative H&E staining images of infected lung tissues after 7 days of treatment. Scale bars, 1 mm and 50 μm (enlarged view). **b,** Representative Wright-stained images of blood in mice with *E. coli* monoinfect after one day of treatment. Scale bars, 20 μm. **c-f**, IL-6 levels (**c**), amount of Gran (**d**) and amount of WBC (**e**) for 1 day in blood. **f**, Bacteria counts in the infected lung after one day with different treatments. Data are presented as mean ± standard deviations from a representative experiment (n = 3 biologically independent samples). P values were analysed by two-way ANOVA with Tukey's multiple comparisons post hoc test. Source data are provided as a Source Data file.

Similarly, a large area of focal infiltration of pink mucus inflammatory cells appeared in the panoramic view of the HE slice in the mouse model of *E. coli* monoinfect pneumonia (Ctrl). And the enlarged image in the group of Ctrl clearly shows the proliferation of alveolar epithelial cells (Supplementary Fig. 25a). In contrast, the lung consolidation decreased in the GN+MV and Gent groups. Meanwhile, the Wright stained blood samples in the treatment group significantly reduced the number of lymphocytes (Supplementary Fig. 25b). And， the amount of IL-6 had decreased in the treatment group (Supplementary Fig. 25c). Besides, Gran (Supplementary Fig. 25d) and WBC (Supplementary Fig. 25e) levels in the treatment group (GNs +MV and Gent) were significantly lower

than that in Ctrl group, indicating that the bacterial infection was restrained in the treatment groups. In addition, the number of *E. coli* in the lungs was significantly reduced in the treatment groups (Supplementary Fig. 25f). Notably, the number of *E. coli* in GN+MV group is almost equal to that of Gent group. These findings indicate that the improvement of GN+MV to pneumonia caused by *E. coli* is similar to that of traditional antibiotics.

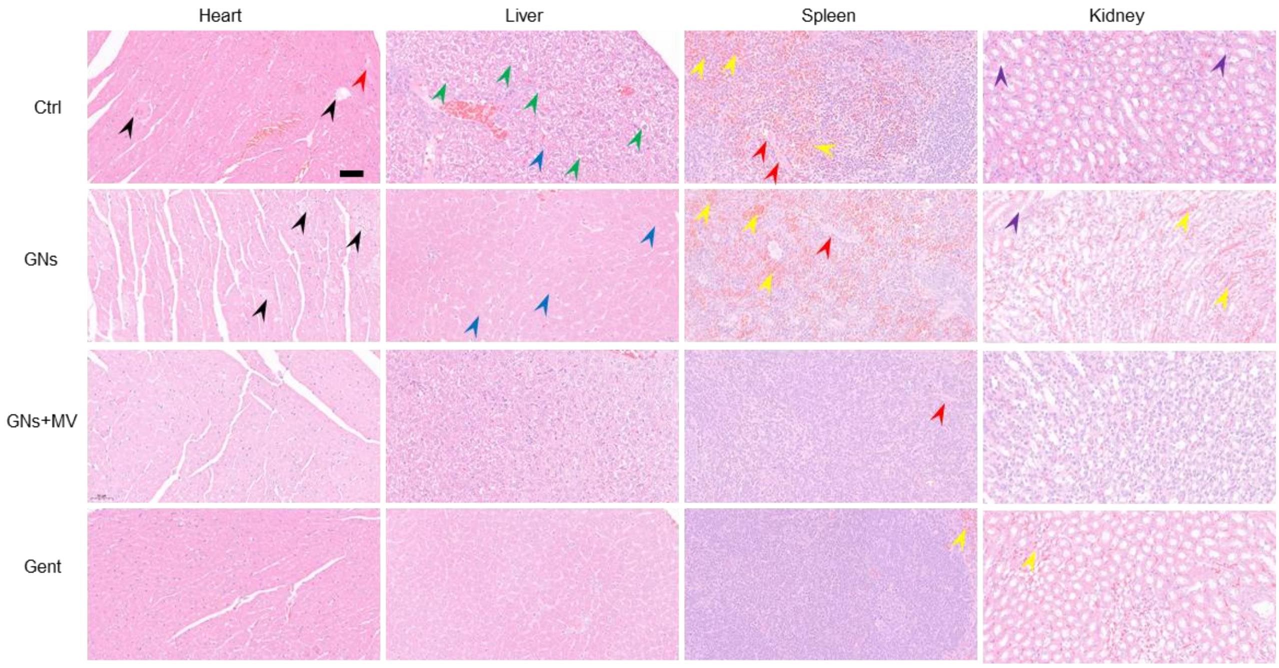

Supplementary Fig. 26 Representative H&E staining images of heart, liver, spleen, and kidney after 7 days post treatment. Scale bar, 50 μm.

Supplementary Table 1 Hight resolution mass spectrometry data of GNs in negative ion mode.

Number	RT (min)	Formula	Name	Measured m/z	Theoretical (m/z).	Area
1	9.4	$C_{39}H_{48}O_9$	10-methoxygambogenic acid	660.3107	660.3298	120810
2	14.1	$C_{33}H_{36}O_8$	Isomorellic acid	560.243	560.2410	676110
3	16	$C_{38}H_{44}O_9$	Gambogic acid	644.3005	644.2985	180614
4	16.9	$C_{38}H_{46}O_8$	Isogambogenic acid	630.3213	630.3193	142341
5	17.8	$C_{38}H_{46}O_8$	Gambogenic acid	630.3213	630.3193	2684136
6	19	$C_{38}H_{44}O_8$	Allogambogic acid	628.3037	628.3036	80904
7	19.4	$C_{38}H_{46}O_7$	Isogambogenin	614.3264	614.3243	974084
8	20.3	$C_{38}H_{44}O_8$	α-Cambogic acid	628.3056	628.3036	4068245
9	23.2	$C_{38}H_{48}O_6$	Desoxygambogenin	600.343	600.3451	401804

Supplementary Table 2 Combination index of GNs combination with MV against *E. coli*.

Factor	F_a	Parameter			CI
		m	D_m	r	
GNs		1.10630	29.7184	0.96698	
MV		2.56030	4.74704	0.97271	
GNs+MV	0.9948				0.10942

The parameter *m*, D*m*, and r represents the slope, antilog of the x-intercept, and the linear correlation coefficient of the median-effect plot accordingly, which signifies the shape of the dose-effect curve, potency (IC_{50}, GNs in mg mL^{-1}; MV in W), and the conformity, respectively.
