# Supplementary material for: Microwave assisted antibacterial action of Garcinia nanoparticles on Gram-negative bacteria
Source: Nat Commun. 2022 May 5;13:2461. doi: 10.1038/s41467-022-30125-w (PMC9072325; doi:10.1038/s41467-022-30125-w)
Supplement: Supplementary file 3 — Description of Additional Supplementary Files [file 41467_2022_30125_MOESM3_ESM.pdf]

Descriptions of additional supplementary Information files

Supplementary Video 1 The conformational change process of OM front view under thermal effect (the group of Ctrl).

Supplementary Video 2 The conformational change process of OM bottom view under thermal effect (the group of Ctrl).

Supplementary Video 3 The conformational change process of OM front view under MV effect (the group of MV).

Supplementary Video 4 The conformational change process of OM bottom view under MV effect (the group of MV).

Supplementary Video 5 The conformational change process of the OM-GNs-1 system under MV effect.

Supplementary Video 6 Dynamic process of GNs-1 during tensile dynamics simulation under MV effect.

Supplementary Video 7 Dynamic process of GNs-1 during tensile dynamics simulation without MV.
